# LInking EDCs in maternal Nutrition to Child health (LINC study) – protocol for prospective cohort to study early life exposure to environmental chemicals and child health

**DOI:** 10.1186/s12889-016-2820-8

**Published:** 2016-02-13

**Authors:** Marijke de Cock, Ilona Quaak, Eva J. Sugeng, Juliette Legler, Margot van de Bor

**Affiliations:** VU University, Health and Life Sciences, Faculty of Earth and Life Sciences, De Boelelaan 1085, 1081 HV Amsterdam, The Netherlands; Brunel University London, Institute of Environment, Health and Societies, room HALB 144, Uxbridge, UB8 3PH UK; VU University, Institute for Environmental Studies, De Boelelaan 1085, 1081 HV Amsterdam, The Netherlands

**Keywords:** Endocrine disrupting chemicals, Developmental origins of health and disease, Early life exposure, Child growth, Neurodevelopment

## Abstract

**Background:**

The presence of chemicals in the environment is ubiquitous. Human biomonitoring studies have shown that various chemicals can be detected in the majority of the population, including pregnant women. These compounds may pass the placenta, and reach the fetus. This early life exposure in particular may be detrimental as some chemicals may disrupt the endocrine system, which is involved in various processes during development. The LINC study is a prospective birth cohort designed to study associations between early life environmental exposures and child health, including growth and neurodevelopment. The purpose of this paper is to give an overview of this cohort.

**Methods and design:**

Recruitment for this cohort has started in 2011 in three Dutch areas and is still ongoing. To date over 300 mother-child pairs have been included. Women are preferably included during the first trimester of pregnancy. Major congenital anomalies and twin births are reasons for exclusion. To assess exposure to environmental chemicals, cord blood, placenta, meconium and vernix are collected. Parents collect urine of the child shortly after birth and breast milk in the second month of life. Exposure to a broad range of environmental chemicals are determined in cord plasma and breast milk. Furthermore various hormones, including leptin and cortisone, are determined in cord plasma, and in heel prick blood spots (thyroxine). Data on anthropometry of the child is collected through midwives and youth health care centres on various time points until the child is 18 months of age. Furthermore cognitive development is monitored by means of the van Wiechen scheme, and information on behavioral development is collected by means of the infant behavior questionnaire and the child behavior checklist. When the child is 12 months of age, a house visit is scheduled to assess various housing characteristics, as well as hand-to-mouth behavior of the child. At this visit exposure of the child to flame retardants (with endocrine disrupting properties) in house dust is determined by means of body wipes. They are furthermore also measured in a saliva sample of the child. Next to these measurements, women receive questionnaires each trimester regarding amongst others lifestyle of the parents, general health of the parents and the child, and mental state of the mother.

**Discussion:**

This study was approved by the medical ethics committee of the VU University Medical Centre. Consent for the infant is given by the mother, who is specifically required to give consent for both herself as well as her child. Results will be published regardless of the findings of this study, and will be widely disseminated among related medical stakeholders (e.g. midwives and pediatricians), policy makers, and the general public.

## Background

Chemicals are used worldwide, making everyday life more comfortable. Their integration into modern day society extends to a level that consumers are often unaware of their presence. They are incorporated in common items, e.g. food packaging materials containing bisphenol a (BPA) and phthalates, which may transfer from the packaging material to the food item itself [1]. Furthermore food products and water may contain pesticides, including those banned from production but persistent in the environment [2]. This ubiquitous presence of chemicals inevitably results in human exposure.

Human biomonitoring studies in the United States detected several chemicals such as perfluorinated alkyl acids (PFAAs) and polychlorinated biphenyls (PCBs) in 90–100 % of the population [3], including pregnant women [4]. They have also been quantified in amniotic fluid [5, 6] and cord blood [7, 8], which indicates that they may pass the placenta and reach the foetus. As the presence of contaminants has also been established in breast milk [9], it is clear that exposure not only starts prenatally, it also continues in early life.

Research has shown that certain compounds may interfere with the function of hormones (endocrine disrupting chemicals, EDCs), including thyroid hormones, estrogen, and testosterone (reviewed by Bergman et al. [10]), which are involved in various processes in adults, but also in brain development early in life [11, 12]. They may also interact with peroxisome proliferator activated receptor α and γ, involved in amongst others adipogenesis [10]. Furthermore, exposure early in life may cause epigenetic modifications, resulting in increased expression or silencing of genes - effects which may be trans-generational [13]. Another point to consider is that exposure early in life may have different effects than exposure in adulthood, as developmental plasticity is much higher in the first period of life [14].

An increasing number of children nowadays face the diagnosis of diabetes type 2 [15], obesity [16, 17], attention deficit hyperactivity disorder [18], autism spectrum disorder [19], and other behavioral disorders. Even though increased risks for each of these health problems have been observed with increased exposure to chemicals [20–22], results are not unambiguous and variations in methodology complicate comparison between different studies. Furthermore, the focus in most observational research has been on non-dioxin-like PCBs and dichlorodiphenyldichloroethylene (DDE), while other compounds such as perfluorinated alkyl acids, and phthalates have been identified as potential health threats as well, but have not yet been investigated as thoroughly.

For most non-communicable diseases important risk factors has been well established, e.g. the role of diet and physical activity in the etiology of obesity and diabetes type 2. However, the increasing prevalence of these diseases in children and their impact on society and health care costs, has created a need to further explore the role of environmental factors. This has led to calls from the European Union to motivate research on this particular topic. As a result, the LInking EDCs in maternal Nutrition to Child health (LINC) study has been set up to investigate health effects of perinatal exposure to a variety of EDCs, and to study determinants of exposure, including parental lifestyle, maternal dietary intake, the residential and occupational environment, and child behaviors. The purpose of this paper is to give an overview of this cohort, for which recruitment has started in 2011, and is currently still ongoing. Study protocols as they currently are used will be reported, including future plans for follow-up. We will furthermore describe the strengths and limitations of this cohort, as well as its’ potential in light of current scientific challenges.

## Methods

**Fig. 1:**
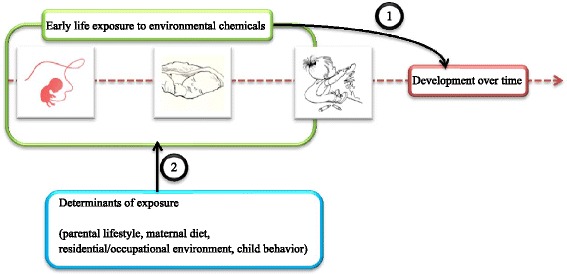
Overview of study aims

The LINC study is a population-based, prospective birth cohort study. It has been initiated to study the effects of perinatal exposure to EDCs on various health outcomes later in life. The general aims of the study are (Fig. 1):

1. To relate early life exposure markers of EDCs with effect biomarkers, child health outcome data and other parameters via multiple regression and multivariate analysis, while taking into account relevant confounders and covariates.
2. To determine factors which are relevant for exposure to EDCs, including, parental lifestyle factors, maternal diet, factors in the residential and/or occupational area, and child behavior types.

### Subjects

**Fig. 2:**
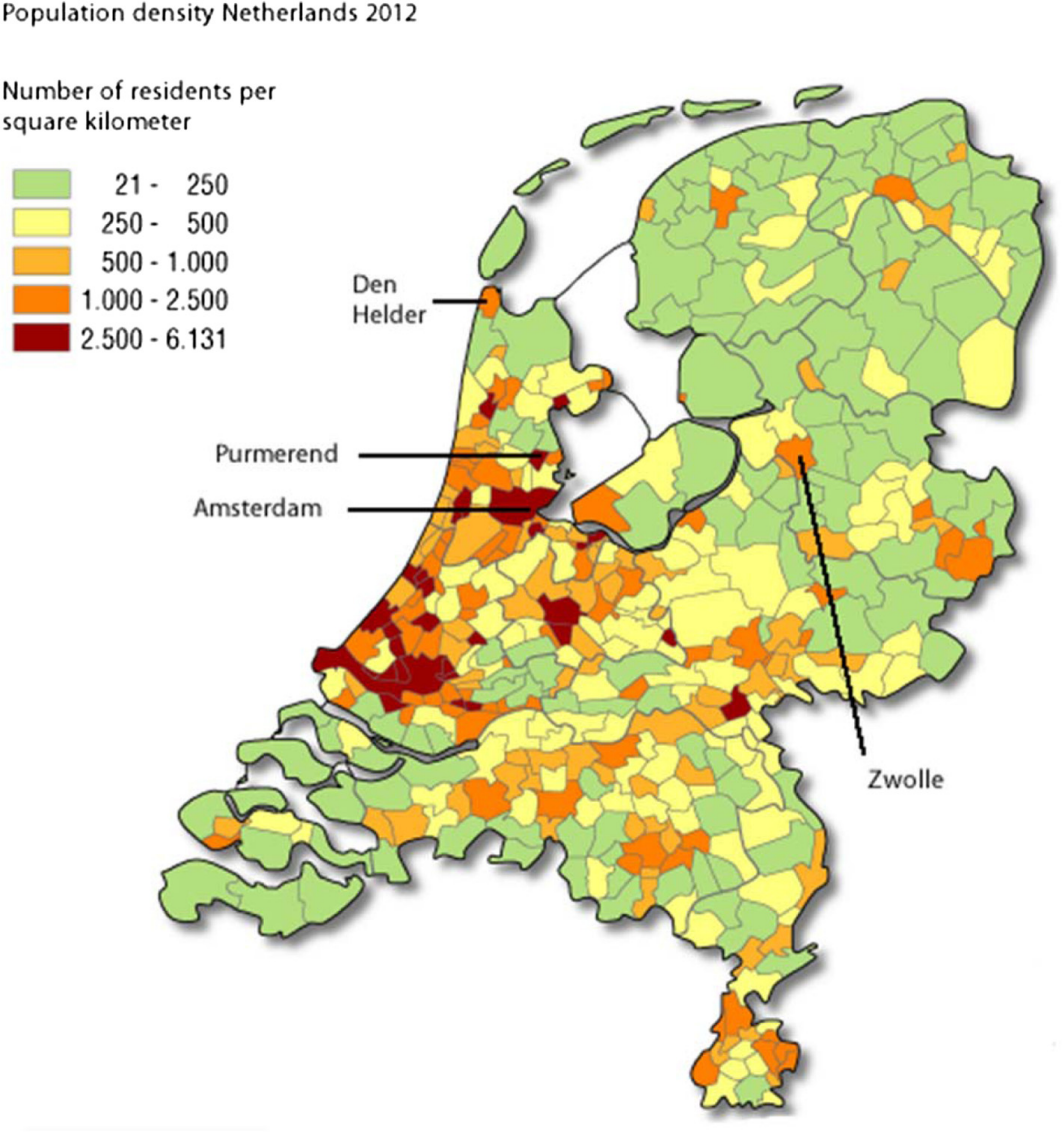
Population density in the Netherlands 2012

All children without major congenital anomalies who are born to women living in the area of Zwolle, Purmerend, or Den Helder (Fig. 2 [23]) are allowed to participate in the cohort. The area of Zwolle in particular was chosen because of the relatively low level of urbanization; about 59 % of the Zwolle city area is used for agriculture. Purmerend and Den Helder differ substantially from Zwolle, both from an environmental and demographic perspective. Purmerend for example is a primarily urban region, and has a higher percentage of population of non-western origins compared to Zwolle (15.1 % vs. 10.0 %). Den Helder on the other hand is characterized by fishery, and a large naval base. Moreover, there is a relatively high number of teen pregnancies (between 0.81–1.53 against 0.6 in the general Dutch population). However, these regional differences offer the opportunity to compare results for different populations.

In the Netherlands, primary care during pregnancy is given by midwives, with obstetricians only involved in complicated pregnancies. Furthermore it is not uncommon for deliveries to occur in the home environment: between 2011 and 2013 approximately 20 % of births were at home [24]. Pregnant women have therefore been recruited through midwifery clinics from 2011 and onwards. Recruitment started in 2011 in Zwolle, after which Purmerend and Den Helder were added as locations. Recruitment in these latter locations is still ongoing, but was finished in Zwolle in 2013. To date over 300 mother-child pairs have been included. Women less than 12 weeks pregnant at their first visit to the clinic are preferred for inclusion, however women may also be included at later stages in pregnancy. They are considered eligible for participation if they are able to fill out questionnaires in Dutch. Decisionally incapacitated subjects are not asked to participate, and twin pregnancies are excluded.

Thus far eight midwifery clinics have been involved in recruitment. Of these, six are located in the area of Zwolle. Midwifery clinics are chosen based on the number of hospitals they cooperate with in the area. If transfer options are limited to one hospital, the clinic is eligible for participation, as this facilitates tracking of child births which do not occur in the home situation. In Zwolle, where recruitment is already finished, about one in fifteen women agreed to participation.

### Follow-up of subjects

**Fig. 3:**
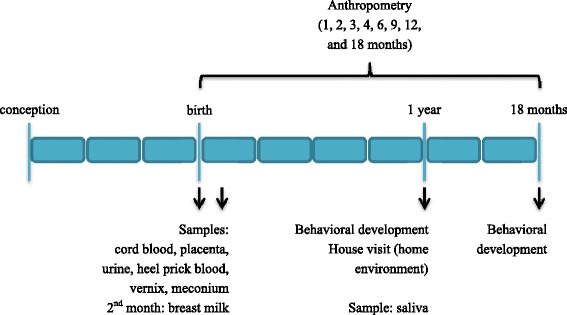
Sample collection and follow-up of main health outcomes within the LINC study

Data from regular care provided by midwives and/or obstetricians is collected for information on women’s health during pregnancy. Further information on the period before and during pregnancy is collected by means of questionnaires, which are sent out to women at inclusion, and at the end of each trimester (4 weeks after birth for the last trimester). Follow-up of the child is done by youth healthcare organizations (at 1, 2, 3, 4, 6, 9, 12, and 18 months of age, according to standardized protocols [25]), which monitor growth and development of children as part of the Dutch health care program. Questionnaires are sent to the mothers when the children are 3, 6, 9, 12, and 18 months of age. Biological samples are collected at birth and in the first 2 months after birth. Furthermore a house visit is scheduled at 12 months of age to make an inventory of the home environment. An overview of the follow-up is given in Fig. 3.

### Measurements

A complete overview of measurements during pregnancy and the first 18 months after birth is given in Table 1.

**Table 1:** Measurements in LINC during pregnancy and the first 18 months after birth

Time	Exposure	Anthropometry	Behavioral and cognitive development	Determinants of exposure	Other (questionnaires)
1^st^ trimester of pregnancy		Weight (mother, and father)		Maternal diet before pregnancy (FFQ)	Demographics
		Length (mother and father)		Occupational and residential exposure	Previous pregnancies, fertility
					Allergies (mother and father)
					Lifestyle
					Mental state, stress (mother)
2^nd^ trimester of pregnancy				Maternal diet during pregnancy (food diary)	Lifestyle
					Mental state, stress
3^rd^ trimester of pregnancy		Weight (mother, 36 weeks of gestation)			General health, medication, family history
					Lifestyle
					Mental state, stress (mother)
					Social support (mother)
Birth	Cord blood	Birth weight			
	Placenta	Head and waist circumference			
	Vernix				
	Heel prick blood				
	Meconium				
	Urine (1^st^ week)				
Month 1		Weight and length	van Wiechen Scheme		
		Head circumference			
Month 2	Breast milk	Weight and length	van Wiechen Scheme		
		Head circumference			
Month 3		Weight and length	van Wiechen Scheme		Infant diet
		Head circumference			Second hand smoke exposure
					Mental state, stress (mother)
					Crying/sleeping pattern
Month 4		Weight and length	van Wiechen Scheme		
		Head circumference			
Month 6		Weight and length	van Wiechen Scheme	Occupational and residential exposure	Infant diet
		Head circumference			Second hand smoke exposure
					Mental state, stress (mother)
					Social support (mother)
					Crying/sleeping pattern
Month 9		Weight and length	van Wiechen Scheme		Infant diet
		Head circumference			Second hand smoke exposure
					Mental state, stress (mother)
					Crying/sleeping pattern
Month 12	Saliva	Weight and length	van Wiechen Scheme	Hand, back, and mouth wipe	Infant diet
		Head circumference	Child behavior (infant behavior questionnaire)	Dust sample	Child allergies
				Occupational and residential exposure	Second hand smoke exposure
				Hand-to-mouth behavior child	Mental state, stress (mother)
					Crying/sleeping pattern
Month 18			van Wiechen Scheme		Second hand smoke exposure
			Child behavior (child behavior checklist)		Mental state, stress (mother)
					Crying/sleeping pattern

All measurements are collected from the child, unless mentioned otherwise

#### Assessment of exposure

The aim is to assess perinatal exposure. For this purpose cord blood is collected from each participant at birth. Blood samples taken from the mother are not collected for the study, as women are allowed to enter the study during any stage of pregnancy. The latest moment of inclusion is just before birth, therefore this moment is most suitable to start collection of samples. Next to that, cord blood is considered a proxy for prenatal exposure and collection is non-invasive for both mother and child. To determine postnatal exposure, mothers are instructed to collect breast milk during 1 week, starting 4 weeks after birth. Use of a breast pump is allowed, and when used, the brand of the pump is provided by the participant. Timing of breast milk collection was decided upon after feedback in a pilot study, in which mothers indicated that collection any earlier after birth was uncomfortable. Furthermore, a small piece from the fetal side of the placenta is collected for epigenetic studies, as well as a sample of vernix and meconium. Parents collect a urine sample of the child during the first week after birth, however we will aim to collect multiple urine samples of the child during early childhood. Finally, at 12 months, a saliva sample of the child is collected.

Exposure to PCB-153, DDE, hexachlorobenzene (HCB), dioxins (CALUX), brominated diphenyl ether (BDE)-47, BDE-99, hexabromocyclododecane (HBCD), perfluorooctanesulfonic acid (PFOS), perfluorooctanoic acid (PFOA), and the secondary DEHP metabolites mono(2-ethyl-5-carboxypentyl) phthalate (MECPP), mono(2-ethyl-5-hydroxyhexyl) phthalate (MEHHP), and mono(2-ethyl-5-oxohexyl) phthalate (MEOHP) will be assessed in cord blood and breast milk. These chemicals will be studied in particular for their suspected obesogenic health effects [26]. Other chemicals will include organophosphates, carbamates, neonicotinoids, pyrethroids, and methylmercury. Some of these compounds, such as the carbamates and neonicotinoids, have not been studied frequently for their health effects in humans. As they are suspected neurotoxicants, they will be predominantly studied in context of child behavioral development. Additionally levels of resorcinol bis(diphenylphosphate) (RBDPP) and bisphenol A bis(diphenylphosphate) (BPA-BDPP) will be determined in the saliva samples as well as the indoor dust samples. These two flame retardants are used as alternatives for deca-BDE in TV or flat screen housings, as well as other electronic equipment. For standardization purposes, the same laboratory is used per compound. Samples which are thus far not used will be stored for additional analyses, and more compounds may be determined as the study progresses. Lab QA/QC procedures have been published previously [27].

#### Health outcomes

There are various parameters early in life which are indicators for development later in life. Birth weight for example is inversely associated with hypertension and type 2 diabetes in adulthood [28, 29], and both high and low birth weight have been associated with obesity [30, 31]. Rapid growth in infancy has been identified as an independent risk factor for childhood obesity [32, 33]. A recent study by Gittner et al. showed that children who were obese at the age of 5 years already had distinct body mass index (BMI) patterns before 12 months of age, with children who had a normal BMI at age five always having a lower BMI from age 6 months and onwards compared to children who were obese at age 5 years [34]. They furthermore showed that BMI patterns over time differed between male and female children. Childhood obesity has also been related to exposure to endocrine disrupting chemicals early in life. Several studies have observed positive associations with BMI for chemicals such as organochlorine pesticides (e.g. DDE, HCB) [7, 35, 36] and PFAAs [37], although results are not unambiguous [38, 39].

Also brain development is regulated and influenced by hormones, and especially thyroid hormones are known to be essential for normal embryonal and foetal neurogenesis [40]. EDCs are known to affect thyroid hormonal function in particular. Therefore disruption of hormonal function during specific time periods important for brain development may have many consequences and may amongst others have adverse effects on neurodevelopment. Positive associations for prenatal EDC exposure and autism spectrum disorders have been reported [41–44], however studies in this area are relatively scarce. For attention deficit hyperactivity disorder, study results seem to be more convincing of an association with early life exposure to endocrine disruptors [20].

Given the physiological processes which may be affected by EDC exposure, it was decided to focus on physical growth as well as neurodevelopment. These outcomes are partially monitored by youth health care centres, which determine child weight, height, waist, and head circumference, and which monitor cognitive development by means of the van Wiechen scheme [45]. Furthermore at 12 months, the Infant Behavior Questionnaire is administered [46], followed by the Child Behavior CheckList (CBCL) at 18 months [47]. An overview of data collected from the child regarding physical growth and behavioral development, and by whom, is given in Table 2.

**Table 2:** Anthropometric and behavioral development data collection by research staff (RS), midwives, and youth health care (YHC)

Parameter	Recorded by			Age child
	RS	Midwives	YHC	
Weight		X	X	Birth, 1, 2, 3, 4, 6, 9, 12, 18 months
Length		X	X	Birth, 1, 2, 3, 4, 6, 9, 12, 18 months
Head circumference		X	X	Birth, 6, 12 months
Waist circumference		X	X	Birth, 6, 12 months
Cognitive development			X	Birth, 1, 2, 3, 4, 6, 9, 12, 18 months
Behavioral development (infant behavior questionnaire, child behavior checklist)	X			12, 18 months

All measurements are performed by trained and experienced staff. Furthermore midwives received a measuring tape as well as strict instructions on how to perform these measurements, including to what precision measurements should be done (e.g. for birth weight: to the nearest 10 g). Weighing scales are provided by the midwife and are calibrated daily. Youth health care organizations received similar instructions as the midwives regarding measurement of weight, length, waist, and head circumference. Furthermore validated questionnaires are used to ensure quality of the data.

#### Determinants of exposure

Exposure to chemicals occurs in part through diet [48]. However, diet is a factor which is relatively easy to control or change and may therefore be a potential pathway through which daily exposure can be decreased. This was for example shown by Rudel et al. [1] who observed a significant reduction after a ‘fresh food’ intervention, which implied that participants followed a 3 day diet containing no canned foods or foods packaged in plastic [1]. We therefore aim to assess the relevance of diet, and certain food groups in particular, in the contribution to the body burden of exposure for our population. Information on maternal dietary habits before pregnancy is collected by means of a food frequency questionnaire [49], which was validated for dioxin intake through diet [50]. This questionnaire is administered at the end of the first trimester. As pregnancy may be a period in which dietary patterns may change because women may want to adopt a more healthy lifestyle, women also fill out a 2-day food diary at the start of the second trimester.

Exposure to flame retardant chemicals specifically occurs mainly through inhalation and ingestion of contaminated house dust. Young children are more exposed to house dust than adults because of their different body composition and their specific behavior. Children have a lower body weight compared to their relative surface area and therefore have a higher intake of flame retardant chemicals. Moreover, children explore their environment with their hands and mouths, performing mouthing behavior, in which objects are touched with the mouth or objects are put into the mouth, increasing exposure to contaminated dust [51, 52]. Finally, children often play close to the floor, enhancing direct contact with house dust.

To assess whether dust is a relevant carrier for flame retardants and if it contributes significantly to exposure during early childhood, home visits are performed when the child is 12 months of age. During these visits a saliva sample is collected from each child, as well as a hand, back, and mouth wipe to assess how much dust accumulates on the surface of the body and can potentially be digested or absorbed through the skin. Furthermore an indoor dust sample from the home is collected. In these samples flame retardants will be measured. Next to that, a questionnaire is administered at the start of the study, and at 6 and 12 months after birth, to inquire about amongst others housing characteristics, furniture and characteristics of electronic devices. Information on cleaning patterns and use of certain products such as personal care products, but also use of pesticides in the house and in the garden, is collected through this questionnaire as well.

#### Effect biomarkers

Associations with health outcomes will be determined for each compound individually. However, the compound by compound approach is not an accurate representation of the mixture of chemicals we are exposed to, and fails to take into account that chemicals may interact and may potentially enhance or diminish the effects they would have individually. It may therefore be useful to study biomarkers of effect. These biomarkers should be able to reflect complex exposures as well as the accumulation of exposures over time [53]. They are furthermore able to relate cause and (health) effect. As we suspect these chemicals to be endocrine disruptors, hormone levels will be included as effect biomarkers.

Cord blood will also be used to determine various hormone levels, including leptin, adiponectin, ghrelin, insulin, cortisone, glucagon, insulin-like growth factor 1, and sex hormone-binding globulin. The Dutch National Institute for Public Health and the Environment is approached in order to obtain data on thyroid hormones (total thyroxine) as measured in the neonatal screening programme.

### Sample size

Sample size was calculated for BMI at 12 months of age. Karmaus et al. [54] observed an increase in BMI of 1.65 kg/m^2^ in offspring of mothers having maternal serum levels of DDE of 1.5 – 2.9 μg/L compared to offspring of mothers having levels of < 1.5 μg/L DDE. Smink et al. [36] measured the HCB in cord blood and related this to BMI at the age of 6 years. Children with levels higher than 1.03 ng/mL HCB had a BMI which was 0.95 kg/m^2^ higher than that of children with levels lower than 0.46 ng/mL HCB. Karmaus et al. did not measure DDE in cord blood and they only measured BMI in the offspring at adult age. Smink et al. on the other hand did measure BMI in children and determined exposure to HCB in cord blood. Therefore we considered a difference in BMI of 1.0 kg/m^2^ relevant.

Previous measurements of BMI in 12 month old Dutch children [55], showed that the variation in BMI among those children is 1.36 kg/m^2^ (1 SD). Furthermore, based on data on DDE levels that already have been obtained in a Belgian cohort [56], we expect that about 30 % of our sample will have DDE levels of < 1.5 μg/L in cord blood.

In order to determine the required sample size for the present study, we entered two rows of data in Microsoft Excel (one with 30 zero’s and one with 70 ones to resemble the estimated distribution of DDE levels dichotomised at 1.5 μg/L). Thereafter we simulated BMI scores to these rows. For the 30 observations, we sampled from a population with a mean BMI of 17.1 (average BMI of boys and girls aged 12 months [55]) and SD of 1.36 and for the 70 observations we sampled from a population with a mean BMI of 18.1 and SD of 1.36. The mean BMI of this latter population is the mean BMI in Dutch boys and girls at the age of 12 months, increased with 1 BMI point. For both groups we selected a sample that resembled the population parameters and we entered those data into SPSS 17. In the following step we conducted a regression analysis with the group variable as independent and BMI scores as outcome. This linear regression analysis resulted in an r^2^ of 0.11. Finally, this r^2^ was used to conduct a sample size calculation for multiple linear regression analysis in STATA 10.0. Assuming a change in r^2^ by adding DDE levels to the model of 0.11, an alpha of 0.05, a power of 0.80 and 10 variables in the final model, the sample size needed is 42. A more conservative estimate of change in r^2^ of 0.05 resulted in a sample size of 88.

The drop-out rate in the study of Karmaus et al. was 55 % [54]. Koletzko et al. [57] followed infants from birth until they were 2 years of age. The drop-out rate was approximately 40 % during the course of that study. To ensure the highest possibility of good statistical power, the sample size in the present study for this outcome is set to *N* = 200. However, experience has indicated that from all participants, only 40 % provides a complete set of body samples. Moreover, from many participants, the volume of the cord blood is too low to be used for measurement of the selected chemicals. For this reason, we would like to extend the sample size to 500 in order to have samples available for 200 participants. Data from participants for whom no samples have been collected, will be used for other purposes than environmental chemical and health outcome associations. Currently over 300 mother-child pairs have been included, which is comparable to several other studies conducted on early life environmental chemical exposure and neurodevelopment in children [58–61].

### Statistical analyses

For all analyses effect modification of the outcome by gender will be checked, as the included chemicals disrupt the endocrine system, and in particular steroid hormones. Linearity of exposure markers and outcome will furthermore be checked due to suspected nonmonotonic dose-response associations [62]. Next to that, various methods will be considered for dealing with exposure values below the limit of quantification (LOQ), including replacement by LOQ/√2 [63], and multiple imputation [64].

In order to quantify the relation between exposure and BMI at the age of 12 and 18 months, we will use linear multilevel (mixed) models. These models take into account the dependency of repeated measures within the individuals. For each compound a separate model will be composed for weight, height, BMI, and head circumference. Exposure quartiles, time, and gender will be added to the models as fixed effects and a random effect will be added for subject. Various covariates, described in 2.5.1, will be tested and included in the model if significant (change in β-coefficient > 10 %).

To study the relationship between exposure and behavior at the age of 18 months, linear regression analyses will be conducted. Both syndrome and DSM-oriented scales from the CBCL will be used for analysis. Syndrome scales are: Emotionally Reactive, Anxious/Depressed, Somatic Complaints, Withdrawn, Attention Problems, Aggressive Behavior, and Sleep Problems. DSM-oriented scales are: Affective Problems, Anxiety Problems, Pervasive Developmental Problems, Attention Deficit/Hyperactivity Problems, and Oppositional Defiant Problems. The overall scales ‘Externalizing’ and ‘Internalizing’, and the total score were also used.

#### Covariates

Potential covariates were selected based on literature. Weight and length of both parents are measured by the midwife at inclusion, approximately 10–12 weeks in pregnancy. Measurement of maternal weight is repeated at 36 weeks of pregnancy to determine gestational weight gain. Gestational age is determined by means of ultrasound. Birth weight is measured by a midwife or a nurse and is obtained from registries of the midwives.

Questionnaires are administered at inclusion and the start of the second trimester to collect information on education (having a bachelor or master degree, yes or no), birth date of the mother, parity, and maternal smoking (yes or no) and alcohol intake during the first trimester (drinks per week). At inclusion, fish intake (grams per day) is determined by food frequency questionnaire which includes both frequencies and portion sizes [49]. One month after birth another questionnaire is administered to inquire on the use of medication during pregnancy, including folic acid intake (yes or no).

## Discussion

### Ethics and safety considerations

Conduction of this study will be according to the principles of the Declaration of Helsinki (version October 22nd, 2008) and in accordance with the Medical Research Involving Human Subjects Act (WMO). This study was approved by the medical ethics committee of the VU University Medical Centre.

The target group of this research is infants aged 0 – 18 months. Consent for the infant is given by the mother, who is specifically required to give consent for both herself as well as her child. Furthermore it will be stressed that participants may leave the project at any time if they desire to do so and that neither withdrawal nor the decision not to participate will affect the care provided to them by midwives or youth health care.

The LINC study is a non-invasive study. The majority of the data will be collected through regular health care (midwives, obstetricians and youth health care). Furthermore, questionnaires will be administered, cord blood, placentas, breast milk, urine, saliva, vernix and meconium will be collected. In addition, hand-wipes, mouth-wipes, and back-wipes will be collected from each child, and waist circumference and head circumference will also be measured in children. All these measurements are non-invasive.

### Dissemination

Results will be published in international peer reviewed journals and multiple doctoral theses, regardless of the findings of this study. Furthermore they will be presented at national and international scientific conferences.

Translation of results to society is considered high priority as this will create knowledge and awareness amongst various stakeholders. We aim to share study results with professional groups related to the target population involved in the cohort, i.e. pregnant women and children. These professional groups include midwives, obstetricians, and pediatricians, but other stakeholders may be involved as the study progresses. We will furthermore disseminate results to the general public as well as policy makers.

### Strengths and limitations

The LINC study is a prospective birth cohort, which is one of the main strengths. Data of the children within the study is already collected very early during pregnancy and though current follow-up is planned until the children are 18 months of age, long-term follow-up is required to proper study associations with growth and behavioural development in children. Approval for extension of follow-up until the children are 48 months of age has already been given, but it is likely that follow-up will be extended to later ages. Furthermore a large variety of data is collected, focussing on multiple aspects of child health including growth, behavior, and nutrition, as well as factors in the direct environment of the child such as parental wellbeing, health, lifestyle, and residential environment.

Biological samples are collected at various time points to complete the information on health and development from each child. A broad range of exposures is assessed within these samples, taking into account already frequently studied compounds such as PCB-153, DDE, and dioxins, but also relatively new groups of chemicals such as perfluorinated alkyl acids, phthalates, carbamates, and pyrethroids. This facilitates comparison with previous studies but also adds to the body of knowledge on less frequently studied compounds. Studies which have assessed such a broad range of environmental chemicals in a variety of samples from a prospective mother-child cohort have thus far been few. We will furthermore aim to look further than the compound-by-compound approach and to look at chemical mixtures, amongst others by studying biomarkers of effect.

The population of the LINC study is very homogeneous, which is an advantage as associations are less likely to be confounded by demographic or socio-economic factors. Extrapolation of results to the general population or other specific groups should be done with caution. Another point is that a relatively small number of respondents is included in the cohort. About one in fifteen pregnant women agreed to participation. A large proportion of the women declined to participate due to the study duration and the anticipated burden of the tasks associated with the study during pregnancy and after birth - periods which were already perceived as quite intense by most potential participants. However, as this is a prospective birth cohort, no selections in study population are made. All women are eligible for participation, unless they are unable to fill out the Dutch questionnaires. These cases have thus far been extremely rare.

Efforts are made to keep drop-out rates as low as possible, amongst others by regularly sharing results of the study and translating these to daily life applications. However, due the duration of the study for each participant, loss to follow-up is inevitable. Thus far 67 participants dropped-out, which is approximately 22 %. This is lower than expected from other studies, and this percentage of drop-out was taken into account in the power calculations. For publications participants included in the study will be compared to participants who were excluded or dropped-out. Next to that, the LINC study has been designed in cooperation with other prospective mother-child cohorts within Europe, which also focus on early life environmental chemical exposure. The main reason for doing as such, is to facilitate cooperation for data-analysis. Through these common efforts, sufficient statistical power can be achieved to study the health effects of environmental chemicals.

In conclusion, the LINC study, as a prospective cohort, will provide insight into the effects of early life exposure to EDCs on child health and development, including childhood obesity and neurodevelopmental disorders. Knowledge on the etiology of these childhood disorders is valuable as this may enable more effective prevention and intervention. Our aim is to extend follow-up of our participants throughout childhood and hopefully adolescence and adulthood to further study long-term health effects of environmental exposures.

## Abbreviations

BDE: brominated diphenylether
BMI: body mass index
BPA: bisphenol A
BPA-BDPP: bisphenol A bis(diphenylphosphate)
CBCL: child behavior checklist
DDE: dichlorodiphenyldichloroethylene
DEHP: bis(2-ethylhexyl)phthalate
EDCs: endocrine disrupting chemicals
HBCD: hexabromocyclododecane
HCB: hexachlorobenzene
LOQ: limit of quantification
MECPP: mono(2-ethyl-5-carboxypentyl) phthalate
MEHHP: mono(2-ethyl-5-hydroxyhexyl) phthalate
MEOHP: mono(2-ethyl-5-oxohexyl) phthalate
PCB: polychlorinated biphenyl
PFAAs: perfluorinated alkyl acids
PFOA: perfluorooctanoic acid
PFOS: perfluorooctanesulfonic acid
RBDPP: resorcinol bis(diphenylphosphate)

## References

[1] Rudel RA, Gray JM, Engel CL, Rawsthorne TW, Dodson RE, Ackerman JM (2011). Food packaging and bisphenol A and bis(2-ethyhexyl) phthalate exposure: findings from a dietary intervention. Environ Health Perspect.

[2] Nougadere A, Sirot V, Kadar A, Fastier A, Truchot E, Vergnet C (2012). Total diet study on pesticide residues in France: levels in food as consumed and chronic dietary risk to consumers. Environ Int.

[3] CDC (2009). Fourth report on human exposure to environmental chemicals.

[4] Woodruff TJ, Zota AR, Schwartz JM (2011). Environmental chemicals in pregnant women in the United States: NHANES 2003-2004. Environ Health Perspect.

[5] Luzardo OP, Mahtani V, Troyano JM, Alvarez de la Rosa M, Padilla-Perez AI, Zumbado M (2009). Determinants of organochlorine levels detectable in the amniotic fluid of women from Tenerife Island (Canary Islands, Spain). Environ Res.

[6] Foster W, Chan S, Platt L, Hughes C (2000). Detection of endocrine disrupting chemicals in samples of second trimester human amniotic fluid. J Clin Endocrinol Metab.

[7] Valvi D, Mendez MA, Martinez D, Grimalt JO, Torrent M, Sunyer J (2011). Prenatal concentrations of polychlorinated biphenyls, DDE, and DDT and overweight in children: a prospective birth cohort study. Environ Health Perspect.

[8] Verhulst SL, Nelen V, Hond ED, Koppen G, Beunckens C, Vael C (2009). Intrauterine exposure to environmental pollutants and body mass index during the first 3 years of life. Environ Health Perspect.

[9] Mikes O, Cupr P, Kohut L, Krskova A, Cerna M (2012). Fifteen years of monitoring of POPs in the breast milk, Czech Republic, 1994-2009: trends and factors. Environ Sci Pollut Res Int.

[10] Bergman A, Heindel JJ, Kasten T, Kidd KA, Jobling S, Neira M (2013). The impact of endocrine disruption: a consensus statement on the state of the science. Environ Health Perspect.

[11] Ahmed OM, El-Gareib AW, El-Bakry AM, Abd El-Tawab SM, Ahmed RG (2008). Thyroid hormones states and brain development interactions. Int J Dev Neurosci.

[12] Weiss B (2012). The intersection of neurotoxicology and endocrine disruption. Neurotoxicology.

[13] Zhang X, Ho SM (2011). Epigenetics meets endocrinology. J Mol Endocrinol.

[14] Newbold RR (2010). Impact of environmental endocrine disrupting chemicals on the development of obesity. Hormones (Athens).

[15] Bloomgarden ZT (2004). Type 2 diabetes in the young: the evolving epidemic. Diabetes Care.

[16] Han JC, Lawlor DA, Kimm SY (2010). Childhood obesity. Lancet.

[17] The European Association for the Study of Obesity - Facts & Statistics [http://easo.org/task-forces/childhood-obesity-cotf/facts-statistics/]. Accessed 8 Feb 2016.

[18] Pastor PN, Reuben CA (2008). Diagnosed attention deficit hyperactivity disorder and learning disability: United States, 2004-2006. Vital Health Stat.

[19] ADDM CDC (2012). Prevalence of autism spectrum disorders--Autism and Developmental Disabilities Monitoring Network, 14 sites, United States, 2008. MMWR Surveill Summ.

[20] de Cock M, Maas YG, van de Bor M (2012). Does perinatal exposure to endocrine disruptors induce autism spectrum and attention deficit hyperactivity disorders? Review. Acta Paediatr.

[21] Alonso-Magdalena P, Quesada I, Nadal A (2007). Endocrine disruptors in the etiology of type 2 diabetes mellitus. Nat Rev Endocrinol.

[22] Hatch EE, Nelson JW, Stahlhut RW, Webster TF (2009). Association of endocrine disruptors and obesity: perspectives from epidemiological studies. Int J Androl.

[23] Mulder M (2012). Volksgezondheid Toekomst Verkenning, Nationale Atlas Volksgezondheid.

[24] CBS (2015). CBS statline: bevalling en geboorte: 1989-2013.

[25] NCJ. JGZ-richtlijn Contactmomenten Basistakenpakket Jeugdgezondheidszorg 0-19 jaar. Utrecht: Nederlands Centrum Jeugdgezondheid; 2003.

[26] Legler J, Hamers T, van Eck van der Sluijs-van de Bor M, Schoeters G, van der Ven L, Eggesbo M (2011). The OBELIX project: early life exposure to endocrine disruptors and obesity. Am J Clin Nutr.

[27] de Cock M, de Boer MR, Lamoree M, Legler J, van de Bor M (2014). First year growth in relation to prenatal exposure to endocrine disruptors - a Dutch prospective cohort study. Int J Environ Res Public Health.

[28] Bergvall N, Iliadou A, Johansson S, de Faire U, Kramer MS, Pawitan Y (2007). Genetic and shared environmental factors do not confound the association between birth weight and hypertension: a study among Swedish twins. Circulation.

[29] Hales CN, Barker DJ, Clark PM, Cox LJ, Fall C, Osmond C (1991). Fetal and infant growth and impaired glucose tolerance at age 64. BMJ.

[30] Yu ZB, Han SP, Zhu GZ, Zhu C, Wang XJ, Cao XG (2011). Birth weight and subsequent risk of obesity: a systematic review and meta-analysis. Obes Rev.

[31] Labayen I, Moreno LA, Ruiz JR, Gonzalez-Gross M, Warnberg J, Breidenassel C (2008). Small birth weight and later body composition and fat distribution in adolescents: the Avena study. Obesity (Silver Spring).

[32] Monteiro PO, Victora CG (2005). Rapid growth in infancy and childhood and obesity in later life--a systematic review. Obes Rev.

[33] Odegaard AO, Choh AC, Nahhas RW, Towne B, Czerwinski SA, Demerath EW (2013). Systematic examination of infant size and growth metrics as risk factors for overweight in young adulthood. PLoS One.

[34] Gittner LS, Ludington-Hoe SM, Haller HS (2013). Utilising infant growth to predict obesity status at 5 years. J Paediatr Child Health.

[35] Mendez MA, Garcia-Esteban R, Guxens M, Vrijheid M, Kogevinas M, Goni F (2011). Prenatal Organochlorine Compound Exposure, Rapid Weight Gain and Overweight in Infancy. Environ Health Perspect.

[36] Smink A, Ribas-Fito N, Garcia R, Torrent M, Mendez MA, Grimalt JO (2008). Exposure to hexachlorobenzene during pregnancy increases the risk of overweight in children aged 6 years. Acta Paediatr.

[37] Halldorsson TI, Rytter D, Haug LS, Bech BH, Danielsen I, Becher G (2012). Prenatal exposure to perfluorooctanoate and risk of overweight at 20 years of age: a prospective cohort study. Environ.

[38] Garced S, Torres-Sanchez L, Cebrian ME, Claudio L, Lopez-Carrillo L (2012). Prenatal dichlorodiphenyldichloroethylene (DDE) exposure and child growth during the first year of life. Environ Res.

[39] Pan IJ, Daniels JL, Herring AH, Rogan WJ, Siega-Riz AM, Goldman BD (2010). Lactational exposure to polychlorinated biphenyls, dichlorodiphenyltrichloroethane, and dichlorodiphenyldichloroethylene and infant growth: an analysis of the Pregnancy, Infection, and Nutrition Babies Study. Paediatr Perinat Epidemiol.

[40] Patel J, Landers K, Li H, Mortimer RH, Richard K (2011). Thyroid hormones and fetal neurological development. J Endocrinol.

[41] Miodovnik A, Engel SM, Zhu C, Ye X, Soorya LV, Silva MJ (2011). Endocrine disruptors and childhood social impairment. Neurotoxicology.

[42] Roberts EM, English PB, Grether JK, Windham GC, Somberg L, Wolff C (2007). Maternal residence near agricultural pesticide applications and autism spectrum disorders among children in the California Central Valley. Environ Health Perspect.

[43] Volk HE, Hertz-Picciotto I, Delwiche L, Lurmann F, McConnell R (2011). Residential proximity to freeways and autism in the CHARGE study. Environ.

[44] Windham GC, Zhang L, Gunier R, Croen LA, Grether JK (2006). Autism spectrum disorders in relation to distribution of hazardous air pollutants in the san francisco bay area. Environ Health Perspect.

[45] Boere-Boonekamp MM, Dusseldorp E, Verkerk PH (2009). Onderbouwing van de validiteit van het ontwikkelingsonderzoek bij kinderen van 0 tot en met 4 jaar, het Van Wiechenonderzoek.

[46] Rothbart MK (1981). Measurement of Temperament in Infancy. Child Dev.

[47] Achenbach TM, Ruffle TM (2000). The Child Behavior Checklist and related forms for assessing behavioral/emotional problems and competencies. Pediatr Rev.

[48] Schafer KS, Kegley SE (2002). Persistent toxic chemicals in the US food supply. J Epidemiol Community Health.

[49] van Dooren-Flipsen MMH, van Klaveren JD (1998). ANI-Voedselfrequentie vragenlijst: ontwikkeling vragenlijst naar de inname van vetoplosbare residuen en contaminanten.

[50] Bilau M, Matthys C, Bellemans M, De Neve M, Willems JL, De Henauw S (2008). Reproducibility and relative validity of a semi-quantitative food frequency questionnaire designed for assessing the intake of dioxin-like contaminants. Environ Res.

[51] Groot E, Lekkerkerk C, Steenbekkers LPA (1998). Mouthing behaviour of young children: An Observational study.

[52] Jones-Otazo HA, Clarke JP, Diamond ML, Archbold JA, Ferguson G, Harner T (2005). Is house dust the missing exposure pathway for PBDEs? An analysis of the urban fate and human exposure to PBDEs. Environ Sci Technol.

[53] Silins I, Hogberg J (2011). Combined toxic exposures and human health: biomarkers of exposure and effect. Int J Environ Res Public Health.

[54] Karmaus W, Osuch JR, Eneli I, Mudd LM, Zhang J, Mikucki D (2009). Maternal levels of dichlorodiphenyl-dichloroethylene (DDE) may increase weight and body mass index in adult female offspring. Occup Environ Med.

[55] Fredriks AM, van Buuren S, Wit JM, Verloove-Vanhorick SP (2000). Body index measurements in 1996-7 compared with 1980. Arch Dis Child.

[56] Govarts E, Nieuwenhuijsen M, Schoeters G, Ballester F, Bloemen K, de Boer M (2012). Birth Weight and Prenatal Exposure to Polychlorinated Biphenyls (PCBs) and Dichlorodiphenyldichloroethylene (DDE): A Meta-analysis within 12 European Birth Cohorts. Environ Health Perspect.

[57] Koletzko B, von Kries R, Closa R, Escribano J, Scaglioni S, Giovannini M (2009). Lower protein in infant formula is associated with lower weight up to age 2 y: a randomized clinical trial. Am J Clin Nutr.

[58] Marks AR, Harley K, Bradman A, Kogut K, Barr DB, Johnson C (2010). Organophosphate pesticide exposure and attention in young Mexican-American children: the CHAMACOS study. Environ Health Perspect.

[59] Sagiv SK, Thurston SW, Bellinger DC, Tolbert PE, Altshul LM, Korrick SA (2010). Prenatal organochlorine exposure and behaviors associated with attention deficit hyperactivity disorder in school-aged children. Am J Epidemiol.

[60] Rakyan VK, Chong S, Champ ME, Cuthbert PC, Morgan HD, Luu KV (2003). Transgenerational inheritance of epigenetic states at the murine Axin(Fu) allele occurs after maternal and paternal transmission. Proc Natl Acad Sci U S A.

[61] Ribas-Fito N, Torrent M, Carrizo D, Julvez J, Grimalt JO, Sunyer J (2007). Exposure to hexachlorobenzene during pregnancy and children’s social behavior at 4 years of age. Environ Health Perspect.

[62] Vandenberg LN, Colborn T, Hayes TB, Heindel JJ, Jacobs DR, Lee DH (2012). Hormones and endocrine-disrupting chemicals: low-dose effects and nonmonotonic dose responses. Endocr Rev.

[63] Hornung RW, Reed LD (1990). Estimation of average concentration in the presence of nondetectable values. Appl Occup Environ Hyg.

[64] Rubin DB (1987). Multiple imputation for nonresponse in surveys.

